# Detecting bit-flip errors in a logical qubit using stabilizer measurements

**DOI:** 10.1038/ncomms7983

**Published:** 2015-04-29

**Authors:** D. Ristè, S. Poletto, M.-Z. Huang, A. Bruno, V. Vesterinen, O.-P. Saira, L. DiCarlo

**Affiliations:** 1 QuTech and Kavli Institute of Nanoscience, Delft University of Technology, PO Box 5046, 2600 GA Delft, The Netherlands; 2 Huygens-Kamerlingh Onnes Laboratory, Leiden Institute of Physics, Leiden University, PO Box 9504, 2300 RA Leiden, The Netherlands

## Abstract

Quantum data are susceptible to decoherence induced by the environment and to errors in the hardware processing it. A future fault-tolerant quantum computer will use quantum error correction to actively protect against both. In the smallest error correction codes, the information in one logical qubit is encoded in a two-dimensional subspace of a larger Hilbert space of multiple physical qubits. For each code, a set of non-demolition multi-qubit measurements, termed stabilizers, can discretize and signal physical qubit errors without collapsing the encoded information. Here using a five-qubit superconducting processor, we realize the two parity measurements comprising the stabilizers of the three-qubit repetition code protecting one logical qubit from physical bit-flip errors. While increased physical qubit coherence times and shorter quantum error correction blocks are required to actively safeguard the quantum information, this demonstration is a critical step towards larger codes based on multiple parity measurements.

## Abstract (web summary)

Future quantum computers will employ error correction to protect quantum data from decoherence and faulty hardware. Here, using a quantum processor with five superconducting qubits, the authors demonstrate how to protect one logical qubit from bitflip errors using multi-qubit, stabilizer measurements.

## Graphical abstract

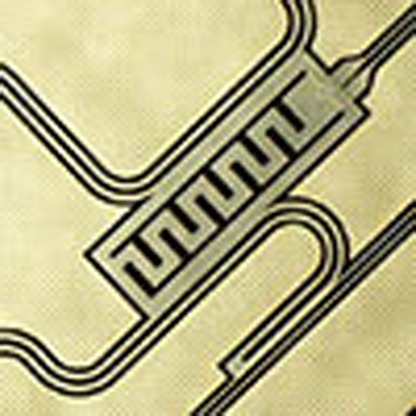

A recent roadmap^1^ for fault-tolerant quantum computing marks a transition from storing quantum data in physical qubits to protection of logical qubits by quantum error correction (QEC)^2^,^3^,^4^,^5^,^6^ as the fourth of the seven development stages. Experimental demonstrations of QEC to date, using nuclear magnetic resonance^7^, trapped ions^8^,^9^, photons^10^, superconducting qubits^11^ and nitrogen–vacancy centres in diamond^12^,^13^, have circumvented stabilizers at the cost of decoding at the end of a QEC cycle. This decoding leaves the quantum information vulnerable to physical qubit errors until re-encoding, violating a basic requirement for fault tolerance. Following steady improvements in qubit coherence, coherent control and measurement over 15 years, superconducting quantum circuits are well poised to face this outstanding challenge common to all quantum computing platforms. Initial experiments using superconducting processors include one round of either bit-flip or phase-flip QEC with decoding^11^, and the stabilization of one Bell state using dissipation engineering^14^. Independent, parallel work^15^ demonstrates the detection of general errors on a single Bell state using stabilizer measurements.

By analogy to the classical repetition code that maps bit 0 (1) to 000 (111), the quantum version maps the one-qubit superposition state *α*|0〉+*β*|1〉 to the entangled Greenberger–Horne–Zeilinger-type (GHZ) state *α*|0_t_0_m_0_b_〉+*β*|1_t_1_m_1_b_〉 of three data qubits (labelled top, middle and bottom)^16^. The stabilizers of this code consist of two-qubit parity measurements described by Hermitian operators *Z*_t_*Z*_m_ and *Z*_m_*Z*_b_. While GHZ-type states are eigenstates of both stabilizers with eigenvalue +1, their corruption by a bit-flip error on one data qubit produces eigenstates with a unique pattern of −1 eigenvalues. Measuring stabilizers can thus discretize and signal single bit-flip errors without affecting the encoded information (that is, the probability amplitudes *α* and *β*). Depending on the error signalled, the logical qubit is transformed to an orthogonal two-dimensional subspace. It is both sufficient and better to keep track of this subspace transformation rather than attempt to return to the original subspace using correcting *π* pulses, which are not perfect and thus introduce errors.

In this work, we construct these stabilizers as parallelized indirect measurements using ancillary qubits, and evidence their non-demolition character by generating three-qubit entanglement from the superposition states. We demonstrate stabilizer-based QEC on the minimal unit of encoded quantum information, a logical qubit, restricting to bit-flip errors.

## Results

### Stabilizer measurements in a superconducting processor

**Figure 1:**
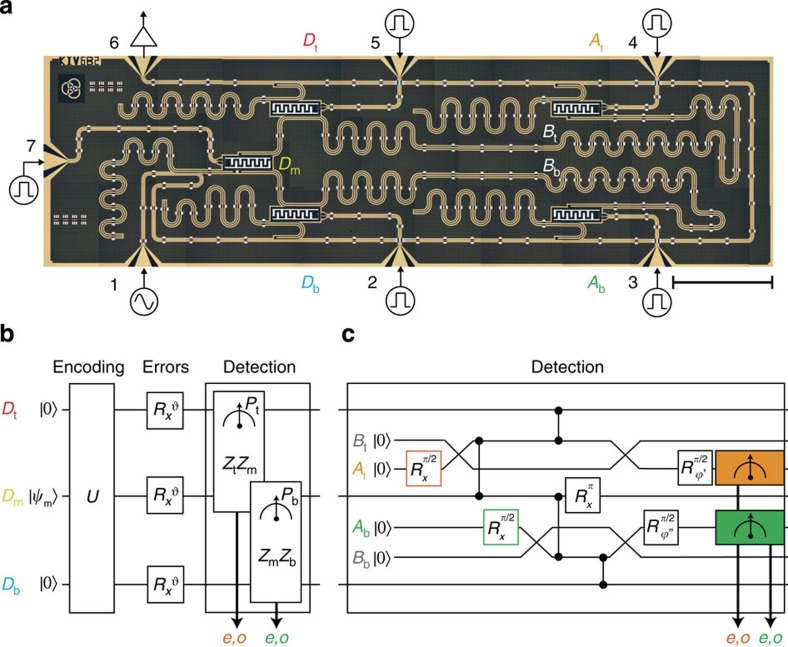
Quantum processor and gate sequence for implementing and characterizing bit-flip QEC by stabilizer measurements. (**a**) Photograph of the processor (scale bar on the bottom-right indicates 1 mm) showing the position and interconnections of data qubits (*D*_t_, *D*_m_ and *D*_b_), ancilla qubits (*A*_t_ and *A*_b_), buses (*B*_t_ and *B*_b_) and dedicated readout resonators. These resonators couple to one common feedline to which all readout and microwave control pulses are applied^18^. Flux-bias lines (ports 2–5 and 7) allow control of the qubit transition frequencies on nanosecond timescale (Supplementary Fig. 1). Details of the processor, including fabrication, parameters and performance benchmarks are provided in Methods and Supplementary Table 1. (**b**) Block diagram for characterizing bit-flip QEC by parallelized parity measurements of pairs (*D*_t_, *D*_m_) and (*D*_m_, *D*_b_). The *D*_m_ state 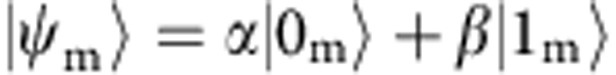 is first encoded into the logical qubit state 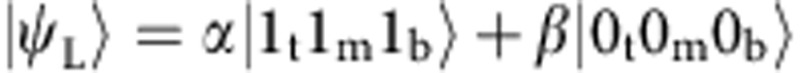. Coherent or incoherent bit-flip errors are then introduced on data qubits with independent single-bit-flip probability *p*_err_. Parallelized *Z*_t_*Z*_m_ and *Z*_m_*Z*_b_ stabilizer measurements discretize these errors and the two-bit measurement result *P*_t_*P*_b_ is interpreted as signalling either no error or error on one qubit. (**c**) Gate sequence implementing the stabilizer measurements by parallelized interaction with ancilla qubits and projective ancilla measurements. Each ancilla is prepared in a superposition state that is transferred to the respective bus with an iSWAP gate (diagonal lines). Consecutive CPHASE gates between each bus and the coupled data qubits (vertical lines) encode the data-qubit parity in the quantum phase of the bus superposition state. The final iSWAP transfers this state to the ancilla, and the latter is then projectively measured in the |±〉 basis. Halfway through the interaction step, a refocusing *π* pulse is applied to *D*_m_ to reduce inhomogeneous dephasing.

This realization of bit-flip QEC with stabilizer measurements employs a superconducting quantum processor with 12 quantum elements (Fig. 1a) exploiting resonant and dispersive regimes of circuit quantum electrodynamics^17^. Three data transmon qubits (*D*_t_, *D*_m_ and *D*_b_) encode the logical qubit. Two ancillary transmons (*A*_t_ and *A*_b_), two bus resonators (*B*_t_ and *B*_b_) and two dedicated ancilla readout resonators are used for the stabilizer measurements. Dedicated readout resonators on data qubits are used to quantify performance (fidelity measures, entanglement witnessing and state tomography). All readout resonators couple to one feedline used for all qubit control and readout pulses. The feedline output couples to a single amplification chain allowing readout of all qubits by frequency-division multiplexing^18^. Ancilla readout fidelity is boosted by a Josephson parametric amplifier^19^ with a bandwidth covering both ancilla readout frequencies (9 MHz apart).

Building on recent developments^20^,^21^, we construct quantum non-demolition stabilizer measurements in a two-step process combining entanglement with ancilla qubits and their projective measurement. Measuring the stabilizer *Z*_t_*Z*_m_ involves an iSWAP gate between *A*_t_ and *B*_t_, two CPHASE gates between *B*_t_ and each of *D*_t_ and *D*_m_, and a final iSWAP transferring the *B*_t_ state onto *A*_t_. These interactions correlate the joint states of *D*_t_ and *D*_m_ with even/odd (*e*/*o*) number of excitations with orthogonal states of *A*_t_. Subsequently, *A*_t_ is measured by interrogating its dispersively coupled resonator. Conveniently, the interaction and measurement steps needed for both the stabilizers can be partially parallelized (Fig. 1c). (Note that a refocusing *π* pulse is applied to *D*_m_ after its interactions to minimize its inhomogeneous dephasing.)

**Figure 2:**
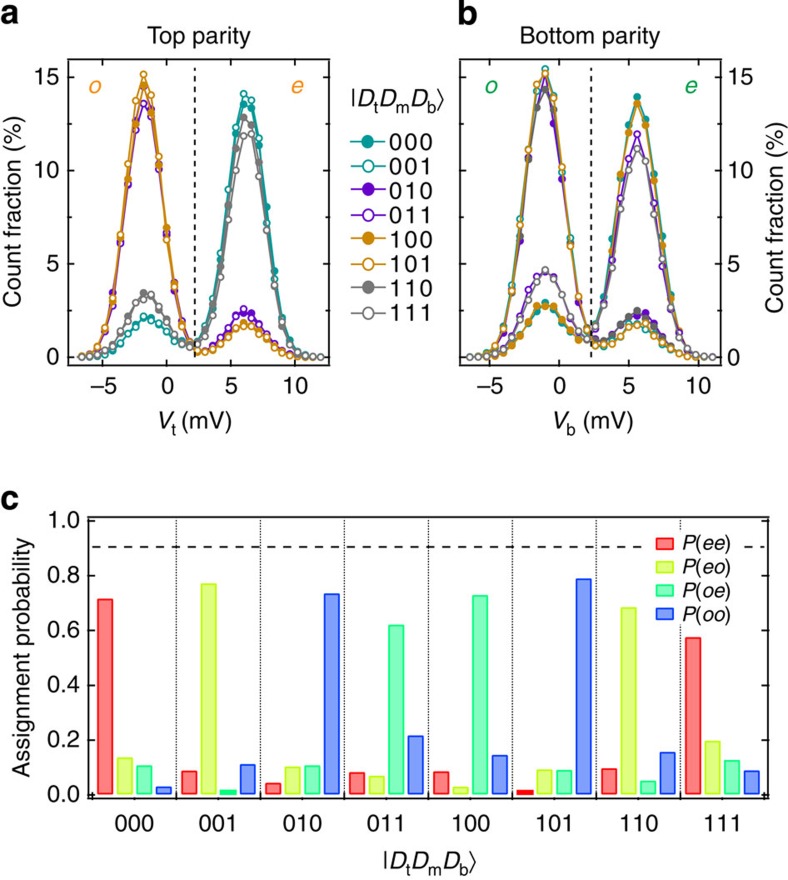
Characterization of stabilizer measurements. Single-shot histograms for top (**a**) and bottom (**b**) ancilla readout signals *V*_t_ and *V*_b_ at the end of the sequence implementing the parallelized stabilizer measurements, with data-qubit computational states as input. The chosen thresholds for discretization of *V*_t_ and *V*_b_ (dashed vertical lines) maximize the parity assignment fidelities. (**c**) Double-parity assignment probabilities for each computational state input. The dashed horizontal line at 0.91 marks the loss of average assignment fidelity exclusively from ancilla readout errors.

We begin characterizing these stabilizer measurements by testing their ability to detect the parities of the computational states |*i*_t_*j*_m_*k*_b_〉, *i*,*j*,*k*∈{0,1}. Because all of these states are eigenstates of *Z*_t_*Z*_m_ and *Z*_m_*Z*_b_, a fixed two-bit measurement outcome *P*_t_*P*_b_∈{*ee*,*eo*,*oe*,*oo*} is expected for each one. Histograms of declared double parities clearly reveal the correlation (Fig. 2). The average assignment fidelity of 71%, defined as the probability of correct double-parity assignment averaged over the eight states, is limited by errors in the interaction step. An upper bound of 91% is set by the combined readout error for the two ancilla measurements (Supplementary Table 1).

### Two- and three-qubit entanglement by stabilizer measurements

**Figure 3:**
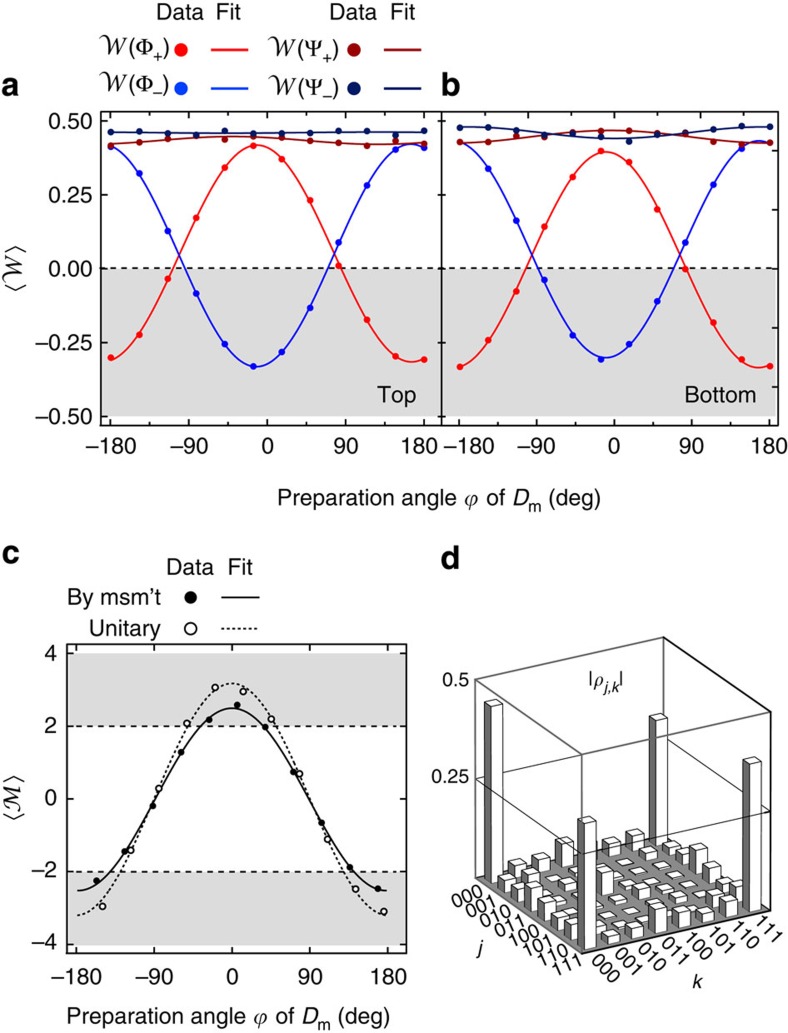
Generation of two- and three-qubit entanglement by stabilizer measurements. Starting with the data qubits in the state 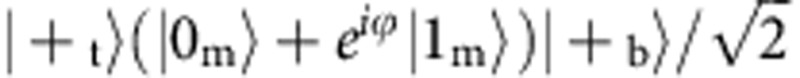, we selectively perform stabilizer measurements by activating the corresponding ancilla, i.e., preparing it in a maximal superposition state. (**a**,**b**), Performing one parity measurement generates entanglement between the paired data qubits. Measured average 
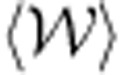
 of the four witness operators 
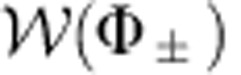
 and 
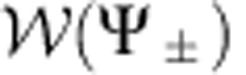
 involving the data qubits paired by activating the top (**a**) or bottom (**b**) ancilla only and postselection on *P*_t_=*o* and *P*_b_=*o*, respectively. Entanglement is witnessed whenever 
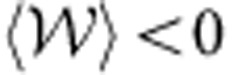
 (shaded area). The weak oscillations in 
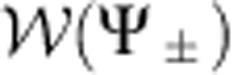
 result from false positives, which we have partially reduced here by postselecting more strongly than the threshold maximizing the average parity assignment fidelity. Standard deviations of 
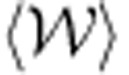
 (∼0.007, smaller than the symbol size) are estimated by bootstrapping 31. (**c**), Measured average of the Mermin operator 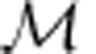 with both ancillas activated and data strongly postselected on *P*_t_*P*_b_=*oo* (black circles). Three-qubit entanglement is witnessed whenever 
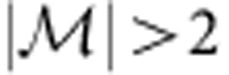
 (shaded area). A stronger violation of the Mermin inequality is observed when targeting the GHZ state 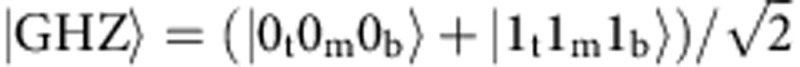 using unitary gates only (white circles). The average standard deviations of 0.1 (encoding by measurement) and 0.08 (encoding by gates) are smaller than the symbol size. (**d**), Tomography (absolute value of the density matrix elements) of the 
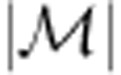
-maximizing state generated by double-parity measurement. The fidelity *F*=〈GHZ|*ρ*|GHZ〉 is 73%. For comparison, targeting this state with gates achieves *F*=82%.

The next test probes the ability of each stabilizer to discern two-qubit parity subspaces while preserving coherence within each. Specifically, we target the generation of two- and three-qubit entanglement (2QE and 3QE) via single and double stabilizer measurements on a maximal superposition state. The gate sequence in Fig. 1c is executed with *D*_t_ and *D*_b_ both prepared in 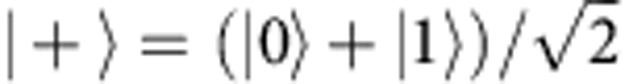 and *D*_m_ in 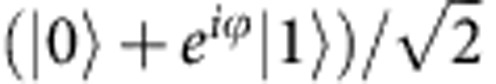. First, we activate one stabilizer by performing the initial *π*/2 rotation only on the corresponding ancilla, and measure the data-qubit-pair witness operators 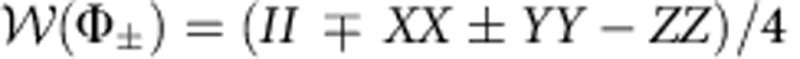, 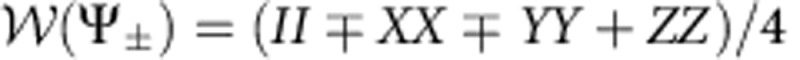 (ref. 22) based on fidelity to even- and odd-parity Bell states, respectively. Each of these operators witnesses 2QE whenever the expectation value 
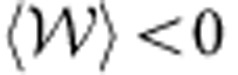
. With postselection on result *o*, either one of 
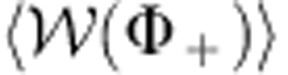
 or 
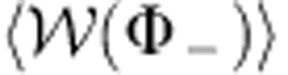
 witnesses 2QE at almost all values of 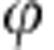 (Fig. 3a,b). A dual result is obtained with postselection on *e*, for which 
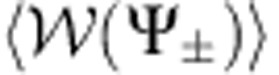
 witness entanglement (data not shown). Note that in both cases the parity of the generated entanglement differs from the detected one due to the refocusing *π* pulse on *D*_m_.

We continue building multi-qubit entanglement by activating both parity measurements and postselecting on the two-bit result (Fig. 3c,d, and Supplementary Fig. 2). Ideally, *P*_t_*P*_b_=*oo* collapses the maximal superposition onto the GHZ-type state 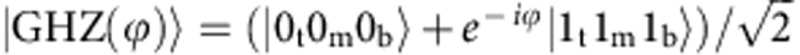. Genuine 3QE is witnessed whenever 
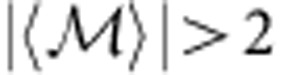
, where 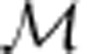 is the Mermin operator *X*_t_*X*_m_*X*_b_−*Y*_t_*Y*_m_*X*_b_−*Y*_t_*X*_m_*Y*_b_−*X*_t_*Y*_m_*Y*_b_ (ref. 23). With postselection on *P*_t_*P*_b_=*oo*, 
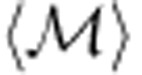
 versus 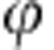 reaches 2.5 (best fit, Fig. 3c). Full state tomography at the optimal 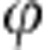 reveals a fidelity 〈GHZ(0)|*ρ*|GHZ(0)〉=73% to the ideal GHZ state (Fig. 3d).

This 3QE-by-measurement protocol can also be used to perform the encoding step of bit-flip QEC. Ideally, the state |+_t_〉 (*α*|0_m_〉+*β*|1_m_〉) |+_b_〉 is mapped onto *α*|1_t_1_m_1_b_〉+*β*|0_t_0_m_0_b_〉 up to the transformation *X*_t_*X*_b_, *X*_t_, *X*_b_, *I* signalled by *P*_t_*P*_b_=*ee*, *eo*, *oe*, *oo*, respectively (the amplitudes *α* and *β*, in addition to the parities, are also exchanged by the refocusing *π* pulse on *D*_m_). Postselection on *P*_t_*P*_b_=*oo* (Supplementary Fig. 3) encodes with 73% fidelity, averaged over the six cardinal input states of *D*_m_, 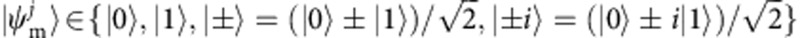). For comparison, implementing the standard unitary encoding^11^,^24^,^25^ using our gate toolbox (Supplementary Fig. 4) achieves 82% average fidelity.

### QEC of bit-flip errors

**Figure 4:**
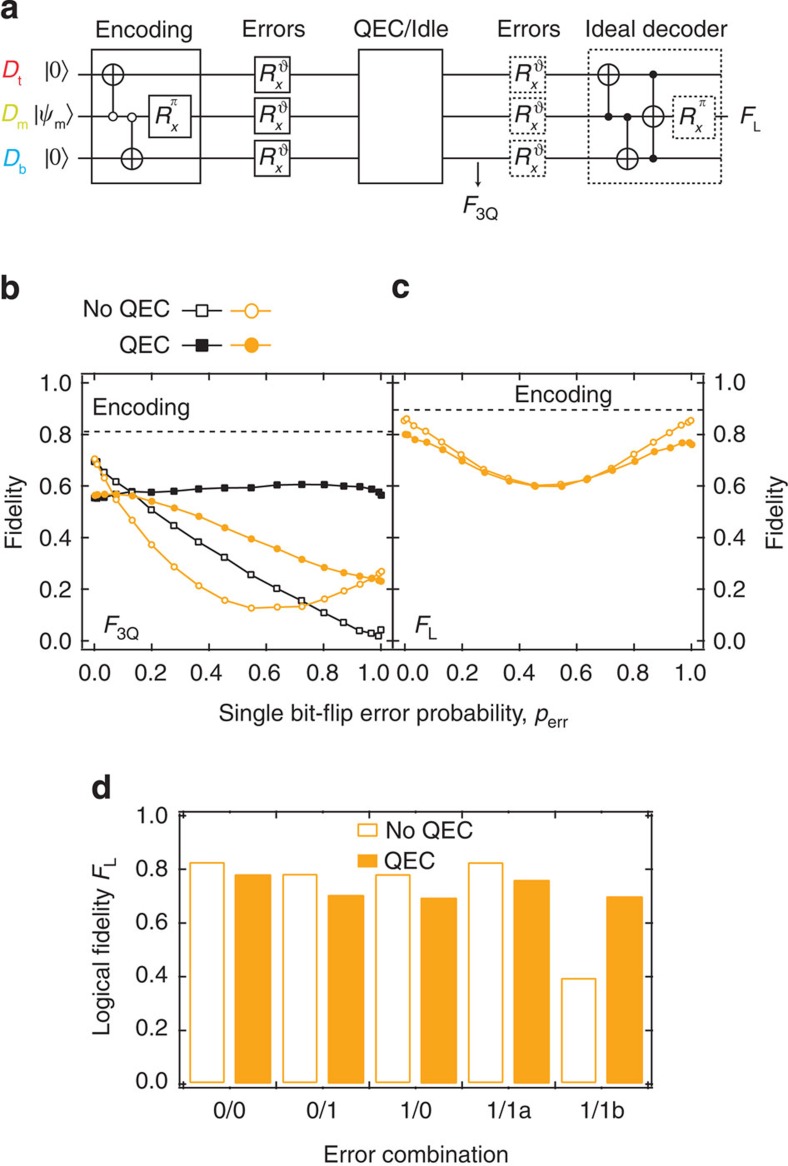
Detection of bit-flip errors. (**a**) Sequence used to assess performance of bit-flip QEC. After encoding by gates, either coherent (*θ*∈[0,*π*]) or incoherent (*θ*=0 or *π*) errors are introduced with single-qubit bit-flip probability *p*_err_. Next, parallelized stabilizer measurements are either performed or replaced by an equivalent idling period. Partial tomography at this point is used to obtain the three-qubit fidelity *F*_3Q_ and the logical fidelity *F*_L_. The calculation of *F*_L_ assumes incoherent second-round errors with the same *p*_err_ and a perfect decoding (dashed boxes). (**b**) Three-qubit fidelity *F*_3Q_ as a function of *p*_err_ with and without QEC under two scenarios: coherent errors applied on *D*_m_ (squares) and on all data qubits (circles). Standard deviations (0.005 for squares, 0.004 for circles) estimated by bootstrapping are smaller than the symbol size. The dashed line indicates the fidelity ceiling imposed by encoding errors. (**c**) *F*_L_ as function of *p*_err_, obtained from the same data as in **b**. The average standard deviation of 0.005 is smaller than the symbol size. The individual contributions of the six cardinal states 
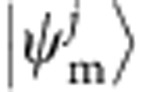
 to *F*_3Q_ and *F*_L_ are shown in Supplementary Fig. 7. (**d**) *F*_L_ for all combinations of one and zero incoherent errors on all data qubits before and after QEC or idling. Error combinations are labelled *m*/*n*, with *m* (*n*) the number of errors before (after) QEC or idling. The case 1/1 is divided in two: errors on the same data qubit (1/1a) or on different qubits (1/1b).

Finally, we use this encoding by gates to demonstrate bit-flip QEC by parallelized stabilizer measurements (Fig. 4a). Bit-flip errors are coherently added via *X* rotations by an angle *θ*, yielding a single-qubit bit-flip probability *p*_err_=sin^2^(*θ*/2) (adding incoherent errors at this stage yields very similar results, see Methods and Supplementary Fig. 5). While the three-bit code is by design only resilient to errors on a single qubit, we also consider the realistic case where such error can occur with the same probability on any of the three qubits. Therefore, we consider two scenarios: the errors added on only one data qubit and the errors added independently on all the three. In both scenarios, we assume no prior knowledge of error probability and literally interpret the stabilizer measurement results as though they were perfect. We first quantify QEC performance using the average fidelity *F*_3Q_ to the ideal three-qubit state accounting for the subspace transformation 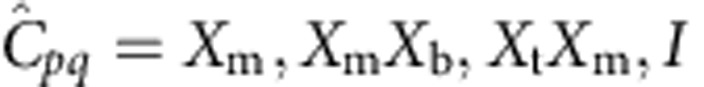 signalled by *P*_t_*P*_b_=*ee*,*eo*,*oe*,*oo* (in order):


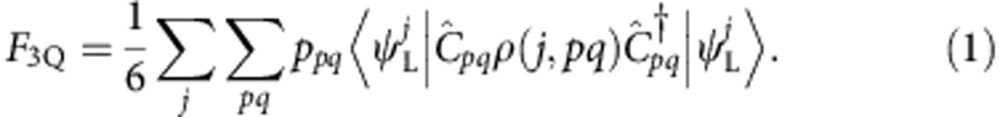


Here, 
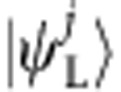
 is the ideal encoded cardinal state, *p*_*pq*_ is the measured probability of *P*_t_*P*_b_=*pq*, and *ρ*(*j*,*pq*) is the experimental *pq*-conditioned density matrix. The near constancy of *F*_3Q_(*p*_err_) with errors on one qubit and the second-order dependence with errors on all three qubits (Fig. 4b) reflect the ability of the stabilizers to discretize and signal single-qubit bit-flip errors without decoding.

To assess the ability of QEC to detect added errors without unfairly penalizing for intrinsic decoherence and encoding errors, we compare *F*_3Q_ with the stabilizer interactions replaced by idling for equal duration (with a refocusing *D*_m_ pulse):


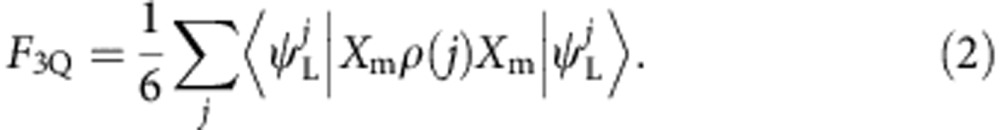


Without QEC, one expects a linear decrease in *F*_3Q_ with errors on one qubit as one bit flip orthogonally transforms the encoded state. The slight curvature observed reflects residual coherent errors in encoding. The non-monotonicity of *F*_3Q_ with errors on all qubits reflects that triple errors perform a logical bit flip, which leaves |+_L_〉 and |−_L_〉 unchanged. Comparing the curves suggests that QEC provides net gains for *p*_err_≳15% in the first case and for *p*_err_≳10% in the second (Fig. 4b).

## Discussion

However, the true merit of QEC hinges on the ability to suppress the accumulation of errors. We believe that a better comparison is the logical state fidelity *F*_L_ following two rounds of errors with QEC or idling in between. *F*_L_ is defined as the fidelity to the initial unencoded *D*_m_ state following an ideal decoder 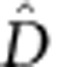 (Fig. 4a) that is resilient to a bit-flip error remaining in any one qubit. For example, with QEC and a second-round error 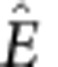,


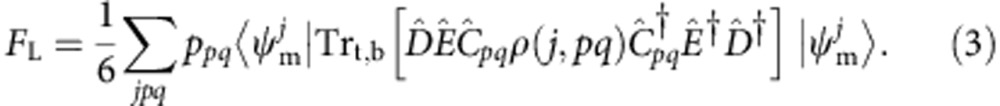


Here we consider the scenario with errors on all three qubits and only incoherent second-round errors. We expect QEC to win over idling in select cases, such as single errors on both rounds but on different qubits, all of which we observe (Fig. 4d and also Supplementary Fig. 6). Weighing in all the possible cases (from 0 to 3 errors in each round) according to their probability, we find that the current fidelity of the stabilizer measurements precludes boosting *F*_L_ for the cardinal states using this quantum repetition code at any *p*_err_ (Fig. 4c). This stricter comparison sets the benchmark for gauging future improvements in QEC.

In summary, we have realized parallel stabilizer measurements with ancillary qubits and used them to perform a quantum repetition code on a superconducting circuit. Stabilizer-based QEC can detect bit-flip errors on data qubits while maintaining the encoding at the logical level, thus meeting a necessary condition for fault-tolerant quantum computing. Evidently, it remains a priority to extend qubit coherence times and shorten the QEC step to boost logical fidelity. In the longer term, parallelized ancilla-based parity measurements as demonstrated here may be used to protect a logical qubit against general errors with a Steane^6^,^26^ or small surface code^27^.

## Methods

### Processor fabrication

The integrated circuit is fabricated on a *c*-plane sapphire substrate. A NbTiN film (80 nm) is reactively sputtered at 3 mTorr in a 5% N_2_ in Ar atmosphere, resulting in a superconducting critical temperature of 15.5 K and normal-state resistivity of 110 μΩcm. This film is e-beam patterned using SAL601 resist and etched by SF_6_/O_2_ RIE to define all coplanar waveguide structures: feedline, resonators and flux-bias lines. The transmon Josephson junctions and shunting interdigitated capacitors are patterned using PMGI/PMMA e-beam lithographed resist and double-angle shadow evaporation of Al with intermediate oxidization. Air bridges are added to suppress slot-line propagation modes, to connect ground planes and to allow the crossing of transmission lines (Supplementary Fig. 8). Bridge fabrication starts with a 6-μm-thick PMGI layer, which is patterned and then reflowed at 220 °C for 5 min, producing a gently arched profile. A second MAA/PMMA resist layer is spun and e-beam patterned to define the bridge geometry. Finally, Ti (5 nm) and Al (450 nm) are e-beam evaporated. The 2 mm by 7 mm chip is diced and cleaned in 88 °C NMP for 30 min.

### Experimental setup

The quantum processor is anchored to the mixing chamber plate of a dilution refrigerator with 15–20 mK base temperature. A detailed schematic of the experimental setup at all temperature stages is shown in Supplementary Fig. 8. The single coaxial line for readout and microwave control has in-line attenuators and absorptive low-pass filters providing thermalization, noise reduction and infrared radiation shielding. Coaxial lines for flux control are broadband attenuated and bandwidth limited (1 GHz) with reactive and absorptive low-pass filters.

### Qubit control

Most microwave pulses for *X* and *Y* qubit rotations have a Gaussian envelope in the main quadrature (5 ns sigma and 20 ns total duration), and a derivative-of-Gaussian envelope in the other (DRAG pulses^28^). Wah–Wah pulses^29^ combining DRAG with sideband modulation are used for *D*_t_ and *A*_b_ to avoid leakage in *D*_m_ and *D*_b_, respectively. Taking advantage of the proximity in frequency between *D*_t_ and *A*_t_, and between *D*_m_ and *A*_b_, we coherently control the five qubits by sideband modulation of three carriers (Supplementary Fig. 8).

Flux pulses for iSWAPs are sudden (12 ns duration), while those for CPHASEs are mostly fast adiabatic^30^ (40 ns). The pulse for CPHASE between *D*_m_ and *B*_t_ is kept sudden (19 ns) to avoid leakage during the crossing of *D*_m_ through *B*_b_. Pulse distortion resulting from the flux control bandwidth is minimized by manual optimization of convolution kernels.

### Qubit readout

The five qubits are readout by frequency division multiplexing^18^. The readout pulses for data and ancilla qubits are separately generated by sideband modulation of the two carriers.

The amplitude and duration of readout pulses are chosen to maximize assignment fidelity. *D*_t_, *D*_m_ and *D*_b_ readout pulses have 1,200, 1,000 and 700 ns duration, respectively. The signal-to-noise boost provided by the Josephson parametric amplifier allows shorter ancilla qubit readouts, 600 ns (550 ns) for *A*_t_ (*A*_b_). The amplified feedline output is split and downconverted with two local oscillators. The two signals are amplified, digitized, demodulated and integrated to yield one voltage for each qubit measured. The low crosstalk between the qubit readouts is evidenced by simultaneous measurement immediately following preparation of the 32 combinations of the five qubits in either |0〉 or |1〉 (Supplementary Fig. 9).

Using the method of ref. 20 based on Hahn echo sequences, we have bound the dephasing of each data qubit induced by the ancilla measurements to <1% (data not shown). Since data-qubit fidelity loss during ancilla measurements is dominated by intrinsic decoherence and our main interest is to quantify the ability of stabilizers to detect the intentionally added errors, we have opted to advance the data qubit measurements, making them simultaneous to those of ancillas (Supplementary Fig. 4).

### Initialization

The four qubits {*D*_t_, *D*_b_, *A*_t_ and *A*_b_} and two buses {*B*_t_ and *B*_b_} are initialized to their ground state by postselection on six measurements performed before any encoding or manipulation protocol. The buses are initialized by swapping states with their coupled ancilla immediately after initialization of the latter. *D*_m_ is initialized by swapping its excitation (∼10%) with that of *B*_b_ (∼1%). The postselected fraction of runs (50–60%) have a residual excitation of 1–2% in every quantum element.

### Gate sequence

Gates are parallelized as much as possible. We note two important exceptions. Because of frequency crowding and the common feedline, pulses targeting one qubit induce ac Stark shifts on untargeted qubits. We serialize single-qubit control to restrict the effect of these shifts to residual phase rotations on unaddressed qubits. Also, the first iSWAP between *B*_t_ and *A*_t_ and CPHASE between *B*_t_ and *D*_m_ (Fig. 1c) are applied before populating *B*_b_ to avoid a strong dispersive shift of *D*_m_. All others iSWAPS, CPHASE gates and ancilla measurements are simultaneous.

### Incoherent errors

We have also tested stabilizer-based QEC with incoherent first-round errors generated using *π* rotations (Supplementary Fig. 5). Following encoding of a *D*_m_ cardinal input state 
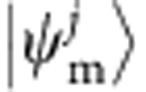
, we apply the eight combinations of error/no error on the three data qubits. We calculate *F*_3Q_ and *F*_L_ for each combination and weigh by the corresponding probability.

## Author contributions

A.B. fabricated the processor, with design input from O.-P.S. and L.D.C. O.-P.S. and V.V. performed the initial tune-up. D.R., M.-Z.H. and S.P. performed measurements and data analysis. S.P., D.R. and L.D.C. prepared the manuscript with feedback from all the other authors. L.D.C. supervised the project.

## Additional information

**How to cite this article**: Ristè, D. *et al.* Detecting bit-flip errors in a logical qubit using stabilizer measurements. *Nat. Commun.* 6:6983 doi: 10.1038/ncomms7983 (2015).

## Supplementary Material

Supplementary Information Supplementary Figures 1-9 and Supplementary Table 1
