# Supplementary material for: Detecting bit-flip errors in a logical qubit using stabilizer measurements
Source: Nat Commun. 2015 Apr 29;6:6983. doi: 10.1038/ncomms7983 (PMC4421804; doi:10.1038/ncomms7983)
Supplement: Supplementary Information — Supplementary Figures 1-9 and Supplementary Table 1 [file ncomms7983-s1.pdf]

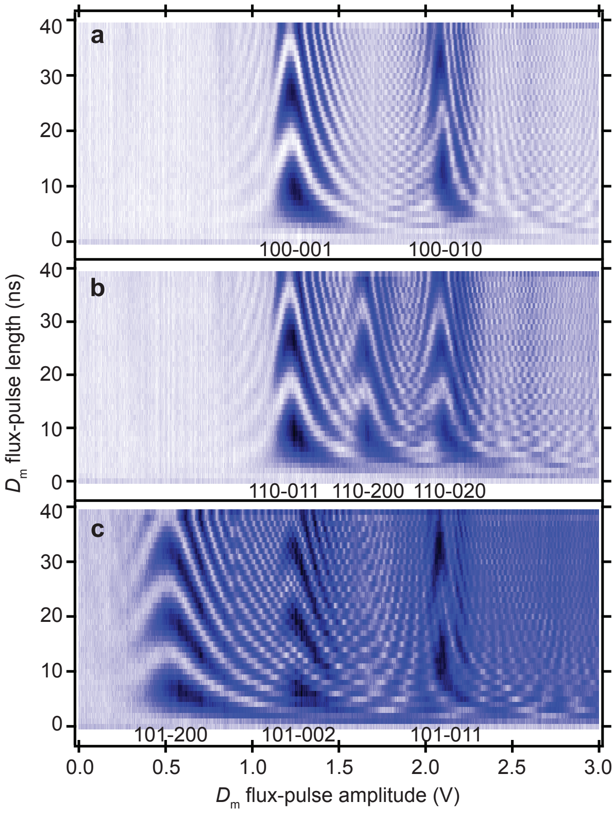

Supplementary Fig. 1: Vacuum Rabi oscillations between D_m and the buses. Coherent oscillations between D_m (initially in $|1\rangle$) and both buses, as a function of flux pulse amplitude and duration. Buses are prepared in $|B_\mathrm{t}B_\mathrm{b}\rangle = |00\rangle$ (**a**), $|B_\mathrm{t}B_\mathrm{b}\rangle = |10\rangle$ (**b**), and $|B_\mathrm{t}B_\mathrm{b}\rangle = |01\rangle$ (**c**). Labels indicate the corresponding transition with notation $|D_\mathrm{m}B_\mathrm{t}B_\mathrm{b}\rangle$

.

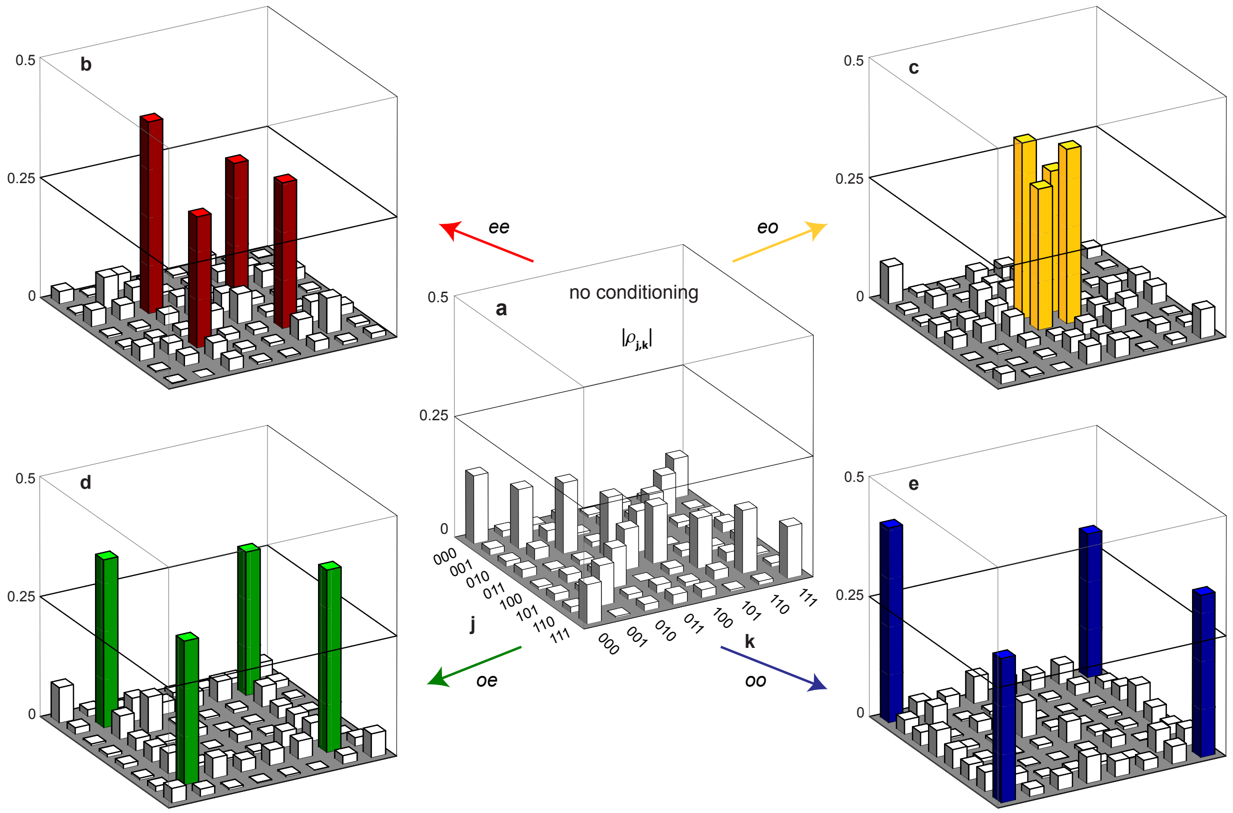

Supplementary Fig. 2: Three-qubit entanglement by parallelized stabilizer measurements on a maximal superposition state. Density-matrix elements (absolute values) of the states obtained by postselection on different stabilizer measurement results: (**a**) No postselection; (**b**) $P_tP_b = ee$, fidelity $\langle\mathrm{GHZ}| X_bX_t\rho X_bX_t |\mathrm{GHZ}\rangle = 61\%$; (**c**) $P_tP_b = eo$, $\langle\mathrm{GHZ}| X_t\rho X_t |\mathrm{GHZ}\rangle = 65\%$; (**d**) $P_tP_b = oe$, $\langle\mathrm{GHZ}| X_b\rho X_b |\mathrm{GHZ}\rangle = 66\%$; (**e**) $P_tP_b = oo$, $\langle\mathrm{GHZ}| \rho |\mathrm{GHZ}\rangle = 67\%$. Note that the parities of the final state differ from the detected ones due to the refocusing π pulse on D_m. In contrast to Fig. 3, conditioning here is performed using the V_t (V_b) threshold maximizing the top (bottom) parity assignment fidelity.

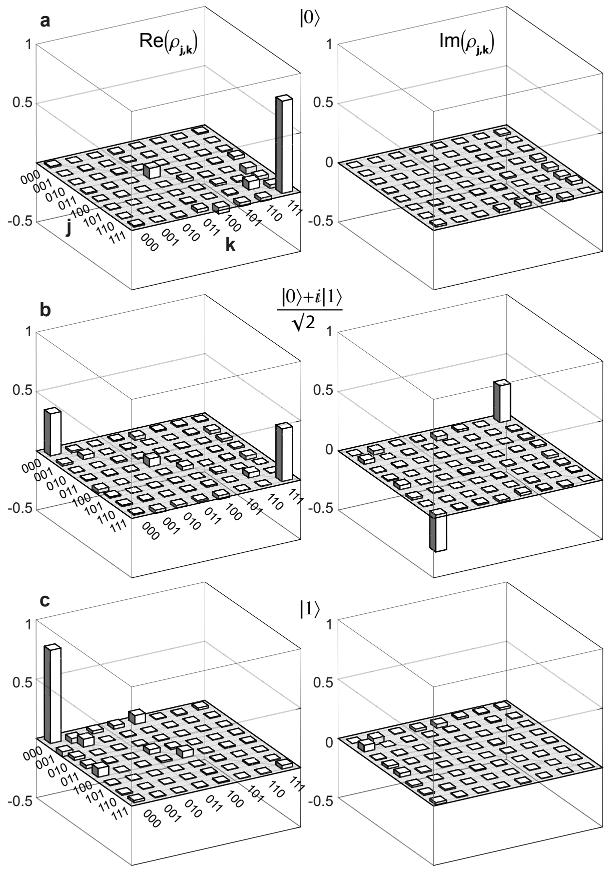

Supplementary Fig. 3: Encoding by measurement. Density-matrix elements (real and imaginary parts) of the state obtained by stabilizer measurements on the state $|+_\mathrm{t}\rangle\,|\psi_\mathrm{m}\rangle\,|+_\mathrm{b}\rangle$ and with strong postselection on $P_\mathrm{t}P_\mathrm{b} = oo$ (as in Fig. 3), with $|\psi_\mathrm{m}\rangle = |0\rangle$ (**a**), $|\psi_\mathrm{m}\rangle = (|0\rangle + i\,|1\rangle)/\sqrt{2}$ (**b**); $|\psi_\mathrm{m}\rangle = |1\rangle$ (**c**). Due to the refocusing pulse on D_m, the state $|0\rangle$ ($|1\rangle$) is encoded in $|1_\mathrm{t}1_\mathrm{m}1_\mathrm{b}\rangle$ ($|0_\mathrm{t}0_\mathrm{m}0_\mathrm{b}\rangle$).

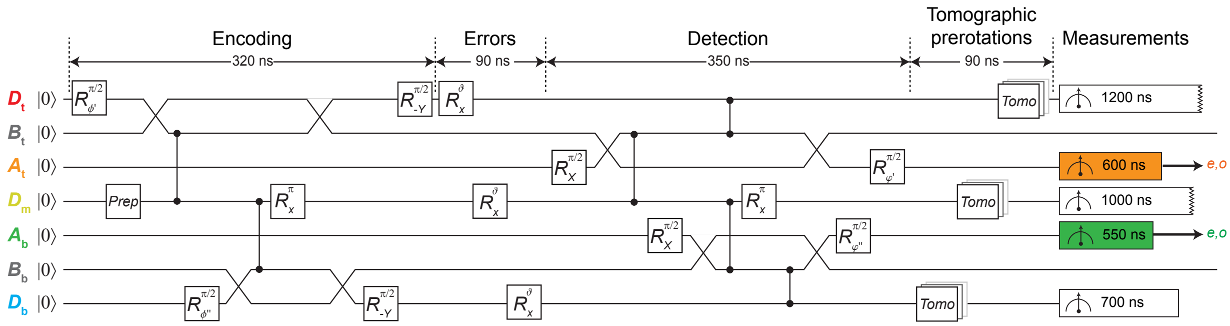

Supplementary Fig. 4: Quantum circuit for QEC characterization. The quantum circuit for QEC characterization has six steps: initialization (not shown), encoding, addition of bit-flip errors, detection, tomographic pre-rotation pulses, and measurements.

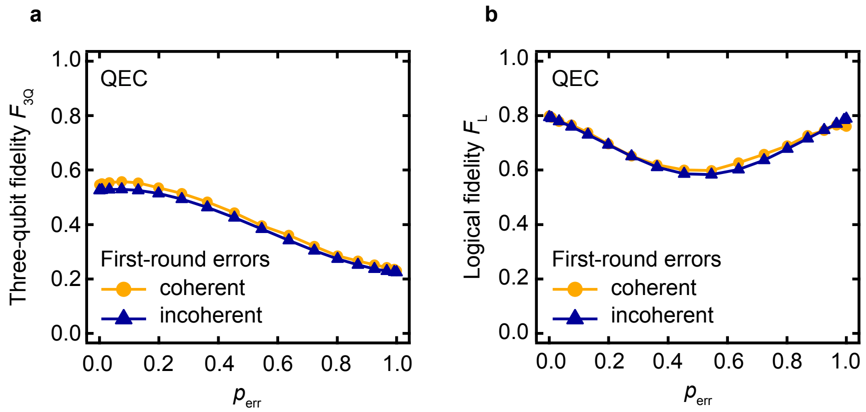

Supplementary Fig. 5: Comparison between coherent and incoherent added errors. Comparison of fidelities F_{3Q} (a) and F_{L} (b) for coherent (circles, same data as in Fig. 4b) and incoherent (triangles) errors applied on the first round on all data qubits with QEC. As expected, the curves closely overlap.

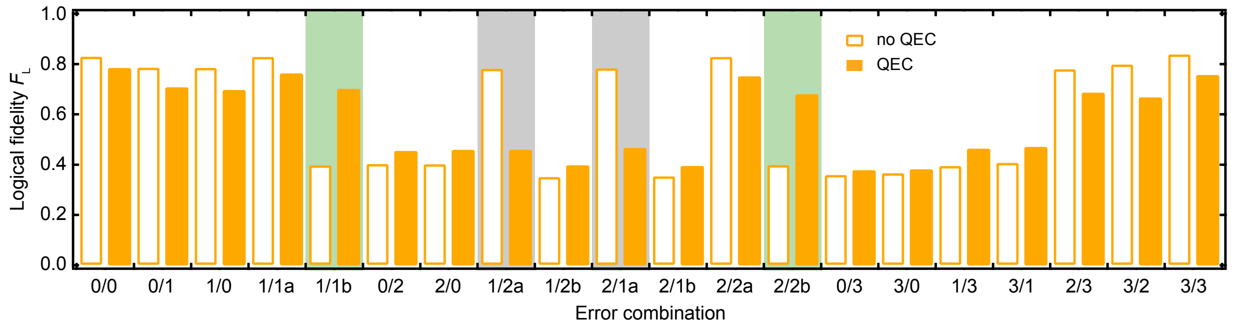

Supplementary Fig. 6: Comparison of logical fidelities F_L for all combinations of first- and second-round errors with and without QEC. Same notation for error combinations as in Fig. 4d. Labels 1/1a and 2/2a indicate first- and second-round errors on the same qubits. Labels 1/2a and 2/1a indicate that one qubit undergoes errors in both rounds. Green regions indicate the error combinations for which QEC is expected to win over idling. Grey regions indicate the opposite. For all other combinations, QEC and idling would ideally tie.

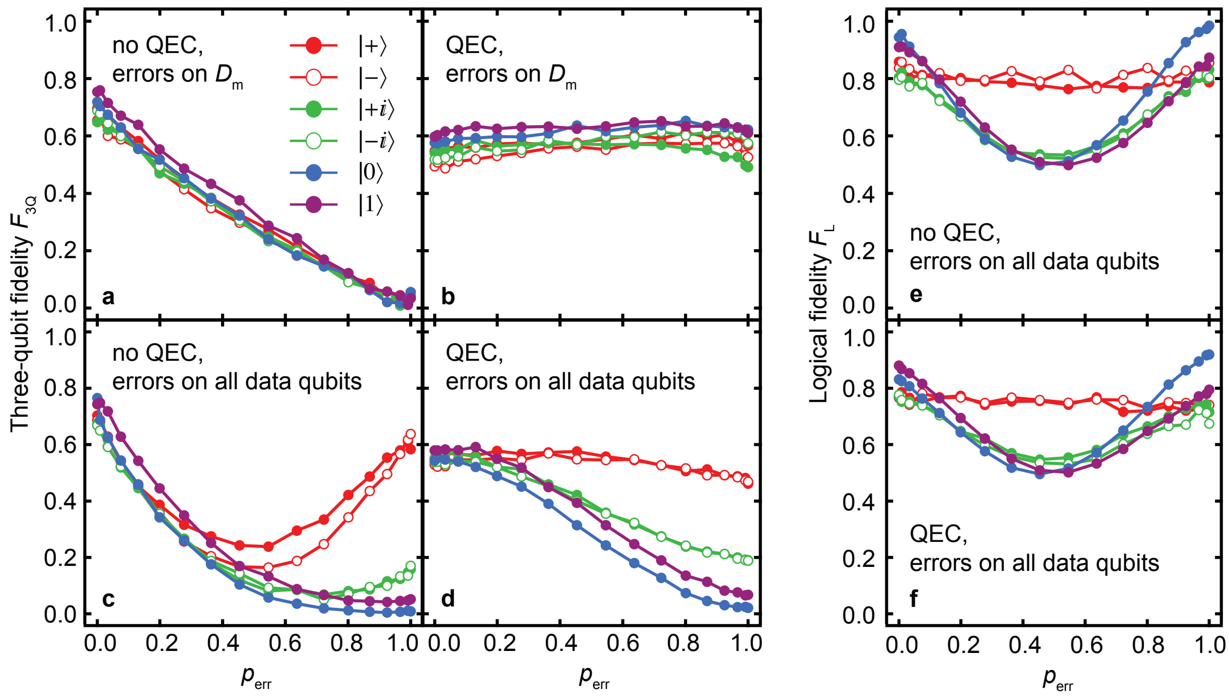

Supplementary Fig. 7: Three-qubit and logical state fidelities for the six cardinal input states of D_m under coherent bit-flip errors. a, b, F_{3Q} for errors on D_m only without and with QEC, respectively. **c, d,** F_{3Q} for errors on all data qubits without and with QEC, respectively. **e, f,** F_L for errors on all data qubits without and with QEC, respectively.

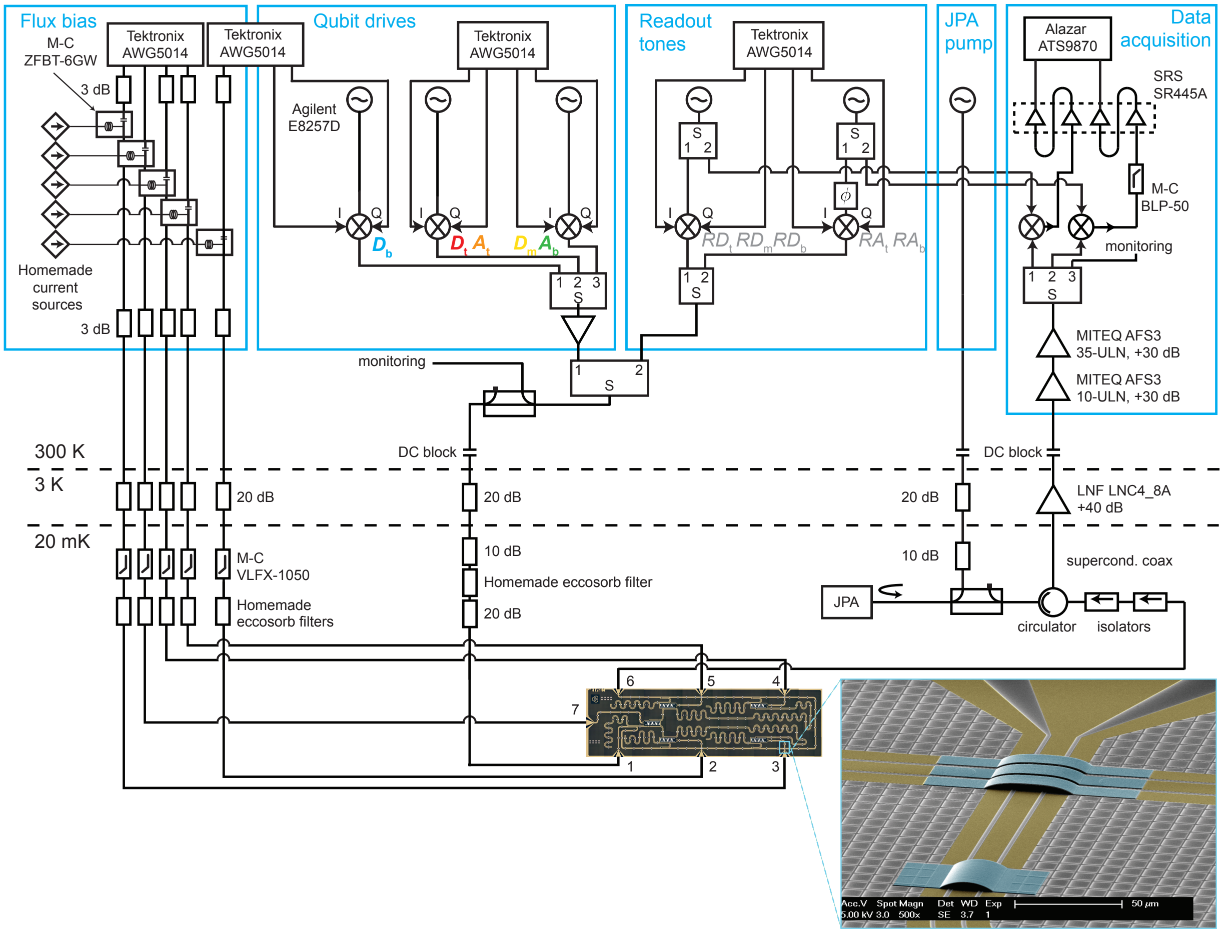

Supplementary Fig. 8: Experimental setup and device details. Complete wiring of electronic components outside and inside the ^{3}He/^{4}He dilution refrigerator (Leiden Cryogenics CF-450). Inset: False-color scanning electron micrograph showing processor details. Coplanar waveguide structures (resonators, feedline, and flux bias lines) are patterned on a NbTiN thin film (gold) on sapphire (gray). Al/Ti air bridges (blue) allow cross-overs between coplanar waveguide transmission lines, interconnections of ground planes, and suppression of slot-line mode propagation.

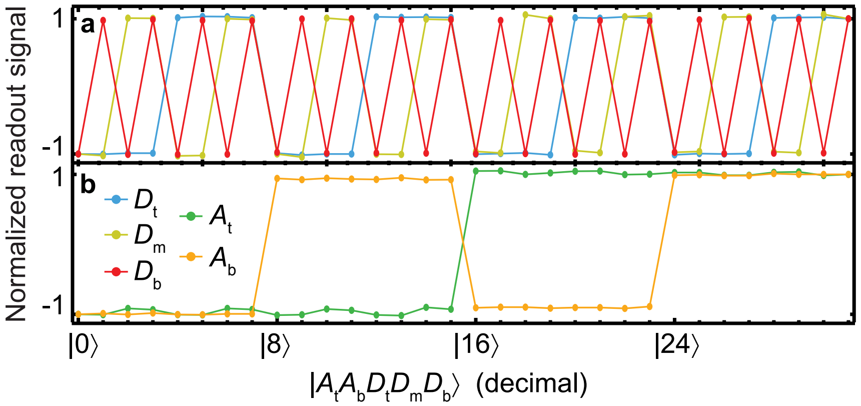

Supplementary Fig. 9: Low-crosstalk simultaneous qubit readouts. Averaged and normalized readouts of the data (**a**) and ancilla (**b**) immediately after preparing the five qubits in the 32 combinations of $|0\rangle$ and $|1\rangle$.

		D_t	D_m	D_b	A_t	A_b	B_t	B_b
max qubit frequency	f_{01}^{max} (GHz)	5.755	6.181	6.788	6.002	6.452	4.80	5.52
qubit operation point	f_{01} (GHz)	5.755	6.065	6.748	5.985	6.452	4.80	5.52
relaxation time	T_1 (μs)	7	13	6	9	10	7	6
pure dephasing time	T_2^* (μs)	3	2	4	3	0.7	13	11
echo time	T_2^{echo} (μs)	7	13	5	4	3		
coupling to buses	$g/2\pi$ to B_t (MHz)	78	48	-	51	-		
	$g/2\pi$ to B_b (MHz)	-	57	58	-	48		
readout resonator	f_r (GHz)	7.599	7.787	7.998	7.095	7.086		
dispersive shift	χ/π (MHz)	-0.6	-0.3	-1.0	-1.6	-2.0		
cavity decay rate	$\kappa/2\pi$ (MHz)	1.7	2.1	1.5	0.9	0.9		
average assignment fidelity		89%	82%	95%	95%	96%		

measurement outcome for top/bottom parity	P_t / P_b
witness operators of two-qubit entanglement	$\mathcal{W}(\Phi_+)$, $\mathcal{W}(\Phi_-)$ (even); $\mathcal{W}(\Psi_+)$, $\mathcal{W}(\Psi_-)$ (odd)
Mermin operator	$\mathcal{M}$
bit-flip error probability	p_{err}

Supplementary Table 1: Summary of the main device parameters and symbols used in the text.
